# Compressive Creep Performances of Dispersion Coated Particle Surrogate Fuel Pellets with ZrC–SiC Composite Matrix

**DOI:** 10.3390/ma18112659

**Published:** 2025-06-05

**Authors:** Qisen Ren, Yang Liu, Runjie Fang, Lixiang Wu, Weiqiang Liu

**Affiliations:** 1 Shenzhen International Graduate School, Tsinghua University, Shenzhen 518055, China; liuweiqiang@mail.tsinghua.edu.cn; 2 China Nuclear Power Technology Research Institute Co., Ltd., Shenzhen 518026, China; liuyang@cgnpc.com.cn (Y.L.); fangrunjie@cgnpc.com.cn (R.F.); wulixiang@cgnpc.com.cn (L.W.)

**Keywords:** accident tolerant fuel, dispersion coated particle fuel, creep, stress exponent, activation energy

## Abstract

Nuclear fuel pellets are subject to stress for long periods during the in-pile operation, and this study on high-temperature creep performance is of great significance for predicting the in-pile behaviors and safety evaluation of fuel elements. In the present study, a mixture of ZrC (50 wt%), SiC (46 wt%), and Si (4 wt%) powder was ball-milled for 24 h and then evaporated to obtain ZrC–SiC composite material. ZrC–SiC composite was adopted as the matrix, with ZrO_2_ surrogate kernel TRSIO particles and dispersion coated particle fuel pellets prepared with different TRISO packing fractions using the Spark Plasma Sintering (SPS) process. This study on compressive creep performances was conducted under a temperature range of 1373–2073 K and a stress range of 5–250 MPa, elucidating the creep behavior and mechanism of dispersed coated particles fuel pellets, and obtaining the variation laws of key parameters such as creep stress exponents and activation energy with TRISO packing fraction. The results showed that creep stress exponents of the surrogate fuel pellets are between 0.89 and 2.12. The activation energies for high temperature–low stress creep (1873–2073 K, 5–50 MPa) are 457.81–623.77 kJ/mol, and 135.14–161.59 kJ/mol for low temperature high stress creep (1373–1773 K, 50–250 MPa). Based on the experimental results, a high-temperature creep model was established, providing a valuable reference for the research and application of a ceramic matrix dispersed with coated particle fuels.

## 1. Introduction

Improving nuclear fuel performance is crucial for reactor design optimization and the safety enhancement of nuclear energy applications. After the Fukushima nuclear accident, enhancing the safety of nuclear reactors has become a major concern for countries around the world. Developing a new type of accident tolerant fuel (ATF) with high intrinsic safety can significantly improve the safety of nuclear power plants and effectively avoid the risk of large-scale radioactive material leakage caused by severe accidents [1,2,3].

Dispersed coated particle fuel (DCPF) draws inspiration from the design of a high-temperature gas cooled reactor nuclear fuel by dispersing multi-layered tri-structural isotropic (TRISO) fuel particles in a ceramic matrix material (such as SiC). The excellent thermal conductivity, high-temperature stability, and oxidation resistance of the matrix material are utilized to improve the thermal conductivity and fission product containment capacity of the fuel pellet.

TRISO particles comprise a spherical fuel kernel and four coating layers of porous pyrolytic carbon buffer, inner dense pyrolytic carbon (IPyC), silicon carbide (SiC), and outer pyrolytic carbon (OPyC) [4,5,6,7,8,9,10,11,12]. TRISO particles are dispersed into a ceramic matrix to make cylindrical fuel pellets, which are then loaded into metal or ceramic composite cladding tubes to form fuel rods. Dispersed coated particulate fuel has excellent accident tolerance performances, which can effectively delay the fuel melting procedure under accident conditions and significantly enhance the ability of radioactivity containment, making it one of the most important potential technologies for the deployment of advanced nuclear energy systems in the future.

In terms of the fuel matrix, SiC is currently the mainstream of research and most of the published studies are focused on the SiC matrix [13,14,15,16]. K. A. Terrani et al. [17] added a small amount (<6%) of Al_2_O_3_ and Y_2_O_3_ into the SiC nano powder, and obtained FCM (fully ceramic micro-encapsulated) fuel samples by hot pressing sintering at 1800–1900 °C and 10–20 MPa. Gyoung Deuk Kim et al. [18] studied the effect of adding a small amount of sintering aid on the SiC matrix and FCM fuel pellets using a hot pressing process. Tan et al. [19] proposed a gel casting process to produce FCM fuel with a high TRISO loading capacity based on SiC. The volume fraction of TRISO particles reached 57%, and the average distance between particles was 52.4 μm, which reduced damage caused by the direct contact between particles. Lei Fu et al. [20] proposed a casting method for preparing FCM fuel pellets with an ordered arrangement of TRISO particles, and successfully prepared samples with TRISO packing fractions ranging from 12.8 to 31.7%. In addition to the SiC matrix, Terrani et al. [21] conducted research on M3 (Metal Matrix Microencapsulated Fuel) using methods such as hot pressing, hot isostatic pressing, and extrusion. Caen Ang et al. [22] proposed using NbC as the matrix for FCM fuel and conducted preparation process research and basic performance testing.

ZrC is an ultra-high temperature ceramic material with a high melting point, high thermal conductivity (especially after irradiation), good irradiation stability, resistance to fission product erosion, low irradiation swelling, and a low neutron absorption cross-section [23]. It is a highly promising candidate material for dispersed coated particle fuel matrix applications. Due to the poor sintering and oxidation resistance of pure ZrC [24], introducing sintering aids and SiC to form a composite material, can effectively promote the sintering densification process and have a positive effect on improving the oxidation resistance of the matrix [25,26].

The nuclear fuel pellets are subject to stress for long periods during the in-pile operation, and this study on high-temperature creep performance is of great significance for predicting the in-pile behaviors and safety evaluation of fuel elements. Currently, there are few reports on the creep performance of dispersed coated particle fuel pellets. Similar research have been performed on U_3_Si_2_ nuclear fuel pellets [27], carbide ceramic such as SiC [28], ZrC [23,29,30], carbide composite [29,31], and high entropy carbide ceramics (HEC) [32,33].

In this study, a small amount of silicon was added to ZrC and then mixed with SiC to form an ZrC–SiC composite matrix. ZrO_2_ was used as the surrogate kernel of the TRSIO particles and the Spark Plasma Sintering (SPS) process was used to prepare dispersed coated particle fuel pellets with different TRISO packing fractions. The impacts of TRISO content, temperature, and stress on the compressive creep performances and behaviors were studied, and a corresponding creep model was established to act as a valuable reference tool for the research and application of ceramic matrix dispersed coated particle fuels.

## 2. Experimental Methods

### 2.1. Sample Preparation

The raw materials used in this study include ZrC powder (0.52 μm, 99.6% purity), Si powder (1 μm, 99.9% purity), SiC powder (0.84 μm, composed of 91 wt% SiC + 5 wt% Y_2_O_3_ + 4 wt% Al_2_O_3_), surrogate TRISO particles (diameter—0.92 mm, ZrO_2_ kernel), and polyvinyl alcohol (PVA).

**Figure 1:**
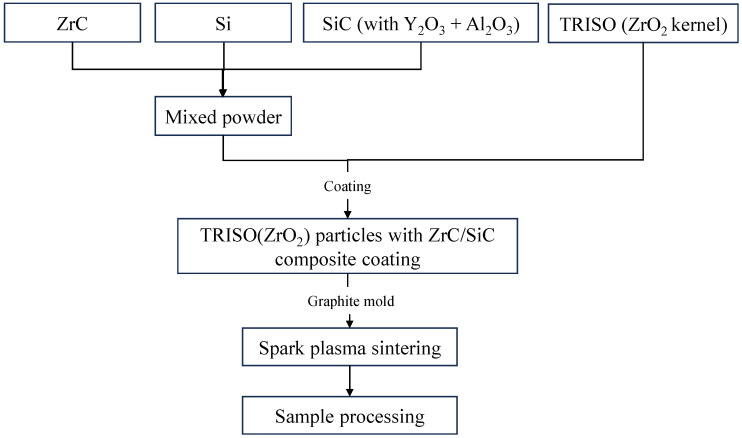
Flowchart of pellet preparation.

The powder was mixed and ball-milled for 24 h with anhydrous ethanol as the liquid and ZrO_2_ as the grinding ball. The milling speed was 15 rpm and the balls-to-powder ratio was 5:1. A rotary evaporator was used to dry the obtained slurries at 60 °C. A certain proportion of mixed powder and TRISO particles was measured, and we used an automatic coating machine to uniformly coat the mixed powder on the TRISO surface. The composition of the mixed powder was 50 wt% ZrC + 46 wt% SiC + 4 wt% Si, and the binder was PVA (Polyvinyl Alcohol) and distilled water in a mass ratio of 1:59. The TRISO particles coated with mixed powder were poured into a graphite mold, and sintered at 1900 °C under 30 MPa for 10 min using the SPS system (FCT h-HPD 10-FL, Rauenstein, Germany). The heating and cooling rates during sintering were both set at 100 °C/min. The flowchart of pellet preparation is shown in Figure 1.

**Figure 2:**
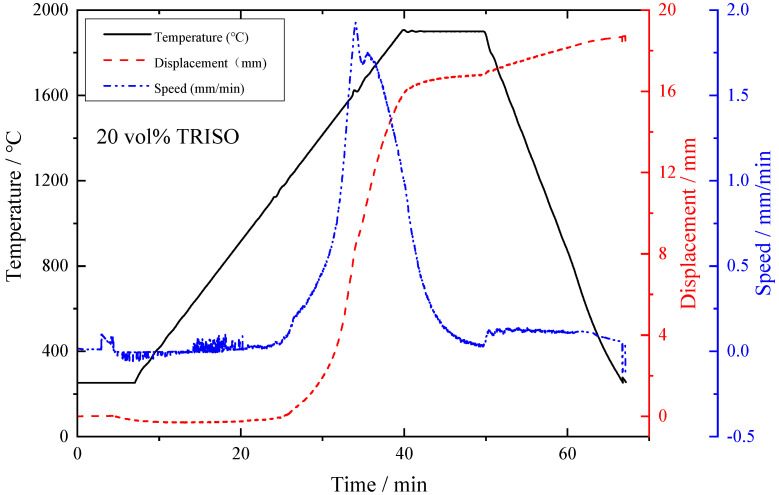
Trend of punch displacement and compression speed over time.

The trend of punch displacement and compression speed over time for 20 vol% TRISO content pellets is demonstrated in Figure 2.

**Figure 3:**
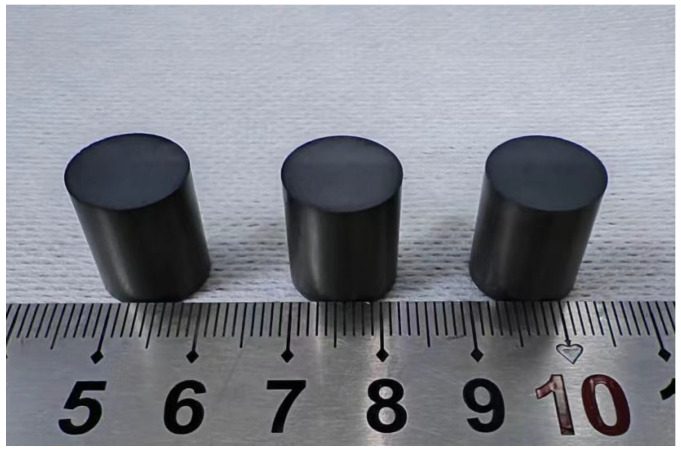
Photograph of dispersed coated particle fuel surrogate pellets.

Figure 3 shows the prepared dispersed coated particle surrogate fuel pellets. The surface of the pellet is smooth, without obvious defects such as cracks or chips. Each pellet is cylindrical and is 12 mm in diameter and 12–17 mm in height. The upper and lower end faces of the samples are precision machined to ensure the parallelism and perpendicularity meet the requirements. The TRISO particle packing fractions in the pellet samples are 0 vol%, 20 vol%, 30 vol%, and 40 vol%, respectively. The appearance of the pellet samples with different TRISO content remains the same.

**Figure 4:**
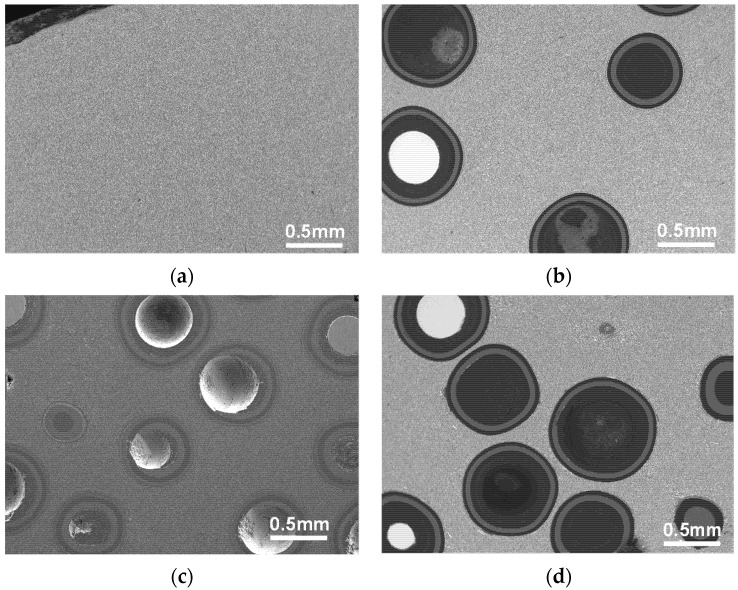
Polished surfaces of surrogate pellet samples with different packing fractions: (**a**) 0%, (**b**) 20%, (**c**) 30%, (**d**) 40%.

The polished surfaces of surrogate fuel pellets were examined by a scanning electron microscopy (Hitachi TM4000PLUS, Tokyo, Japan) using a backscattered electron detector, as shown in Figure 4. Results show that the sintered ZrC–SiC matrix is dense and uniform, and the multi-layer coatings of TRISO particles remain integral. The interfaces between each layer are clear, and there is no particle contact or obvious particle deformation.

**Figure 5:**
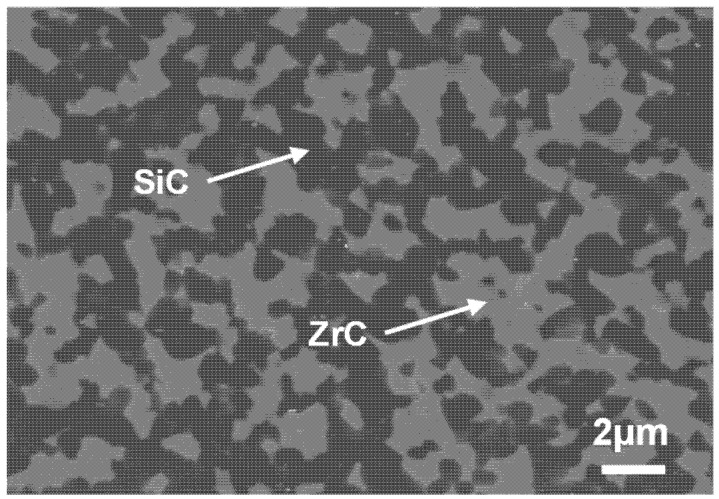
Microstructure of the ZrC–SiC matrix.

Further characterization on the microstructure of the ZrC–SiC matrix was conducted by SEM with a lager magnification (Figure 5). The results showed that the ZrC–SiC matrix had good density and integrity, and that there was a uniform distribution of ZrC and SiC grains.

The densities of sintered pellets with TRISO particle content ranging from 0 to 40 vol% were measured using the Archimedes’ principle. The pellet samples achieved a high density which was greater than 95% T.D., as shown in Table 1.

### 2.2. Compressive Creep Testing Protocol

Compressive creep experiments were carried out on samples with different TRISO particle packing fractions according to the conditions shown in Table 2. In order to study the temperature and stress dependence of pellet creep performance, a step loading method was adopted, and various stress levels were designated at a fixed temperature. Among them, the first three experiment conditions with lower temperatures (1373–1773 K) considered the creep behavior under high stress (50–250 MPa), while the last three experiment conditions with higher temperatures (1873–2073 K) considered the creep behavior under low stress (5–50 MPa). In order to systematically investigate the effect of temperature on creep behavior, this study adopted six temperature levels between 1373 and 2073 K. At lower temperatures (less than 1773 K), a temperature level was set every 200 K. For higher temperature (greater than 1873 K) conditions, the creep deformation of the pellet is significant, and the temperature intervals were proposed to be 100 K to limit the pellet deformation. For conditions with lower temperatures, the holding time under each stress load is ~60 min, while at higher temperatures, the holding time is shortened to ~10 min to avoid deformation and fracture of the samples. The setting of holding times should ensure that the compressive creep of the sample reaches the stable creep stage, thereby obtaining the steady-state creep rate under different temperature and loading stress conditions. The compressive creep experiments were performed using a customized high-temperature creep testing machine in argon atmosphere.

## 3. Results and Discussion

### 3.1. Creep Strain

**Figure 6:**
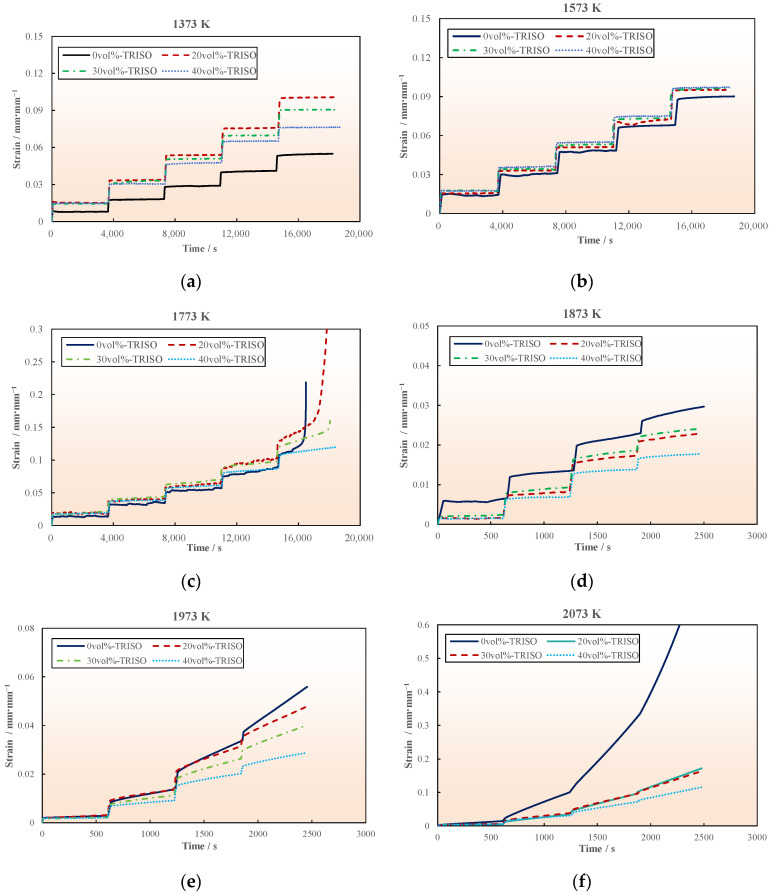
Creep strain curves under different temperatures: (**a**) 1373 K, (**b**) 1573 K, (**c**) 1773 K, (**d**) 1873 K, (**e**) 1973 K, and (**f**) 2073 K.

At lower creep temperatures (1373–1773 K), the instantaneous strain of the pellet is very significant when the loading stress increases, as displayed in the creep strain curve in Figure 6. Subsequently to the instantaneous deformation stage, there is an obvious steady-state creep stage with a low constant strain rate (on the order of 10^−8^–10^−6^ s^−1^). The creep strain of pellet samples with different TRISO contents is primarily the same, with no significant difference. Pellet samples experienced fracture failure at 1773 K when the stress increased to 250 MPa, except for the 40 vol% TRISO sample. The creep fracture time of the pure matrix (0 vol% TRISO) sample is earlier than that of the pellet with TRISO particles, as shown in Figure 6c. The possible reason for this phenomenon is that the TRISO multi-layer coating structure has high strength and is not easily deformed. During the creep experiment, the creep strain generated by axial compressive load mainly comes from the matrix material. The introduction of TRISO particles tends to form a creep reinforcement phase, which contributes to improving the creep resistance of dispersed fuel pellets.

When the temperature further increases to 1873–2073 K, the instantaneous deformation of the sample is not significant. At this time, a noticeable deceleration creep stage can be observed from the creep curve (as shown in Figure 6d–f), mainly because the ceramic pellet sample exhibits plasticity with the increasing temperature. It can be deduced that the creep strain of the pellet sample containing TRISO particles is significantly lower than that of the pure matrix sample, under the same temperature, stress, and creep time conditions. This further indicates that the introduction of TRISO particles has a positive effect on strengthening the high-temperature creep performance of the pellet.

Instantaneous deformation is the elastic response of the specimen to applied loads. As the creep experiments in this study were performed adopting a step loading method, the magnitude of stress increase during each loading is the main factor determining the instantaneous strain. In the experiment at the low temperature stage (1373–1873 K), the stress increased by 50 MPa with each step loading, and the instantaneous strain was more obvious, about 0.01–0.025 mm/mm^−1^. In the experiment at the high temperature stage (1873–2073 K), the stress increase amplitude of each step loading was 10–20 MPa, and the instantaneous strain was relatively small, which is not clearly reflected in the creep curve.

### 3.2. Post-Creep Microstructure

**Figure 7:**
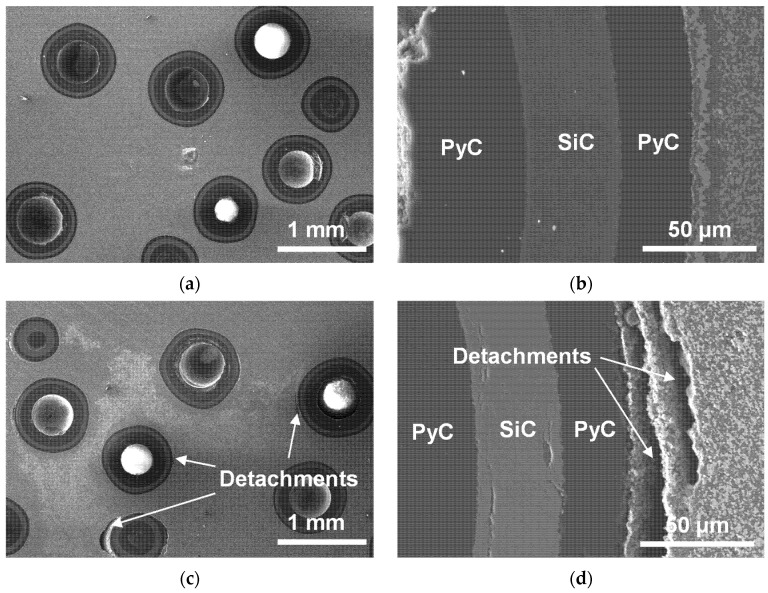
Microscopic morphology of 20% vol TRISO samples after creep at 1373 K/2073 K: (**a**) After 1373 K creep, cross-section of pellet; (**b**) After 1373 K creep, cross-section between TRISO and matrix; (**c**) After 2073 K creep, cross-section of pellet; (**d**) After 2073 K creep, cross-section between TRISO and matrix.

A scanning electron microscopy was used to characterize the microstructure of the samples with 20% vol TRISO content after creep experiments at 1373 K/2073 K (as exhibited in Figure 7). At a lower temperature (1373 K), the pellet creep deformation is small, and the interfaces between TRISO particles and the matrix are well bonded. While at higher temperatures (2073 K), the pellet axial creep deformation becomes significant, and there are visible detachments at the interfaces between TRISO particles and the matrix. The detachments extend along the circumference of TRISO particles, with a width of about 20–100 μm. The possible reason is that during the creep experiment, deformation of the pellet is mainly from the ZrC–SiC matrix, and the TRISO particles hardly deform and instead maintain the initial geometric structure. The mismatch between the two deformations leads to separation at the interface. Overall, the ZrC–SiC matrix and TRISO particles maintain good uniformity and integrity.

### 3.3. Creep Properties

#### 3.3.1. Stress Exponents

The creep strain rate of ceramic materials can be expressed using the following relationship [34]:

(1) $$\dot{\varepsilon }=A\sigma^{n}exp\left(-\frac{Q}{kT}\right)$$

where $\dot{\varepsilon }$ is the steady-state creep strain rate, s^−1^; *A* is the model constant, s^−1^·MPa^−n^; *σ* is the creep stress, MPa; *n* is the creep stress exponent; *Q* is the creep activation energy, kJ/mol; *k* is the Boltzmann constant, 8.314 kJ/mol/K; *T* is the creep temperature, K.

Keeping the creep temperature constant, taking the logarithm on both sides of Equation (1), the expression of the creep stress exponent can be obtained as follows:

(2) $$n=\frac{\ln\left({\dot{\varepsilon }}_{1}\right)-\ln\left({\dot{\varepsilon }}_{2}\right)}{\ln\left(\sigma_{1}\right)-\ln\left(\sigma_{2}\right)}$$

**Figure 8:**
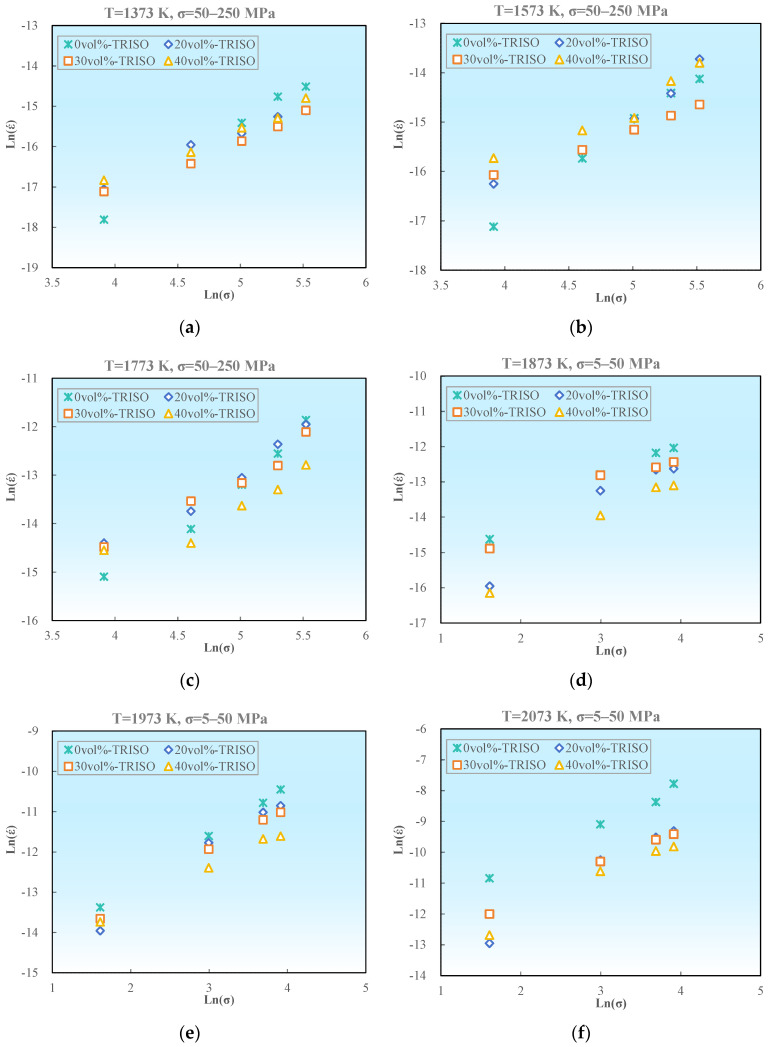
Relationship between creep rate and stress, ln ($\dot{\varepsilon }$)–ln (*σ*): (**a**) T = 1373 K, σ = 50–250 MPa; (**b**) T = 1573 K, σ = 50–250 MPa; (**c**) T = 1773 K, σ = 50–250 MPa; (**d**) T = 1873 K, σ = 5–50 MPa; (**e**) T = 1973 K, σ = 5–50 MPa; (**f**) T = 2073 K, σ = 5–50 MPa.

Based on the aforementioned experimental data, the steady-state creep strain rate and creep stress of pellet samples with different TRISO contents were logarithmically calculated and plotted as ln ($\dot{\varepsilon }$)–ln (*σ*), as demonstrated in Figure 8. The slope of the curve represents the creep stress exponent *n*.

**Figure 9:**
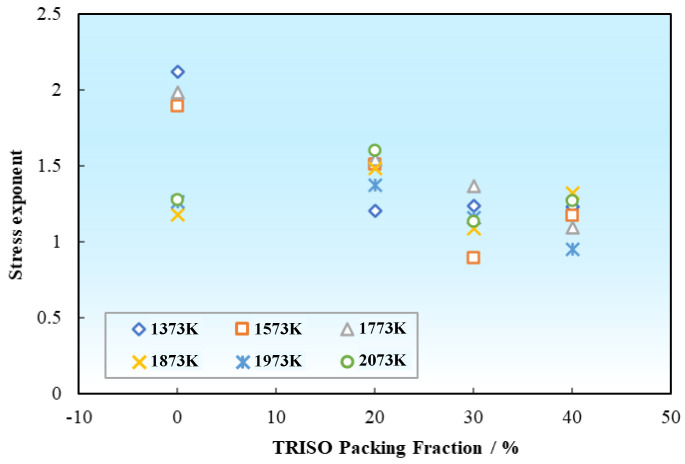
Variation of creep stress exponents with TRISO packing fractions at different temperatures.

The stress exponent *n* of pellet samples with different TRISO contents at different temperatures is shown in Table 3. It can be concluded that for the pure matrix of 0 vol% TRISO sample, the creep stress exponent *n* ≈ 2 in the low temperature range (1373–1773 K), and *n* closer to 1 at the higher temperature range of 1873–2073 K. While for the pellet samples with 20–40 vol% TRISO content, the creep stress exponents are generally around 1, with a slightly decreasing trend with the increase in TRISO content, as displayed in Figure 9. As a whole, both the pure matrix and the pellet samples with TRISO particles have creep stress exponents ranging from 0.89 to 2.12, with no significant difference, indicating that their creep mechanisms are probably similar.

#### 3.3.2. Activation Energy

Keeping the loading stress constant, taking the logarithm on both sides of Equation (1), the expression of creep activation energy can be obtained as follows:

(3) $$Q=-k\frac{\ln\left({\dot{\varepsilon }}_{1}\right)-\ln\left({\dot{\varepsilon }}_{2}\right)}{1/T_{1}-1/T_{2}}$$

**Figure 10:**
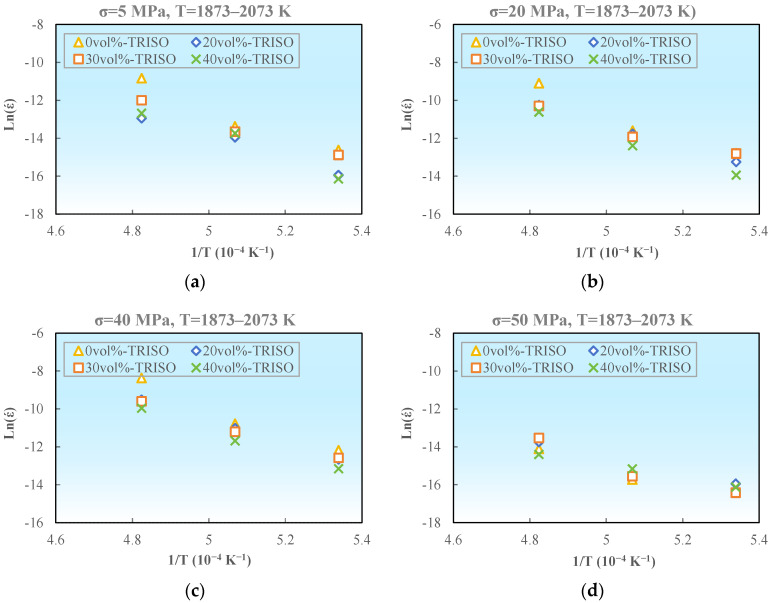

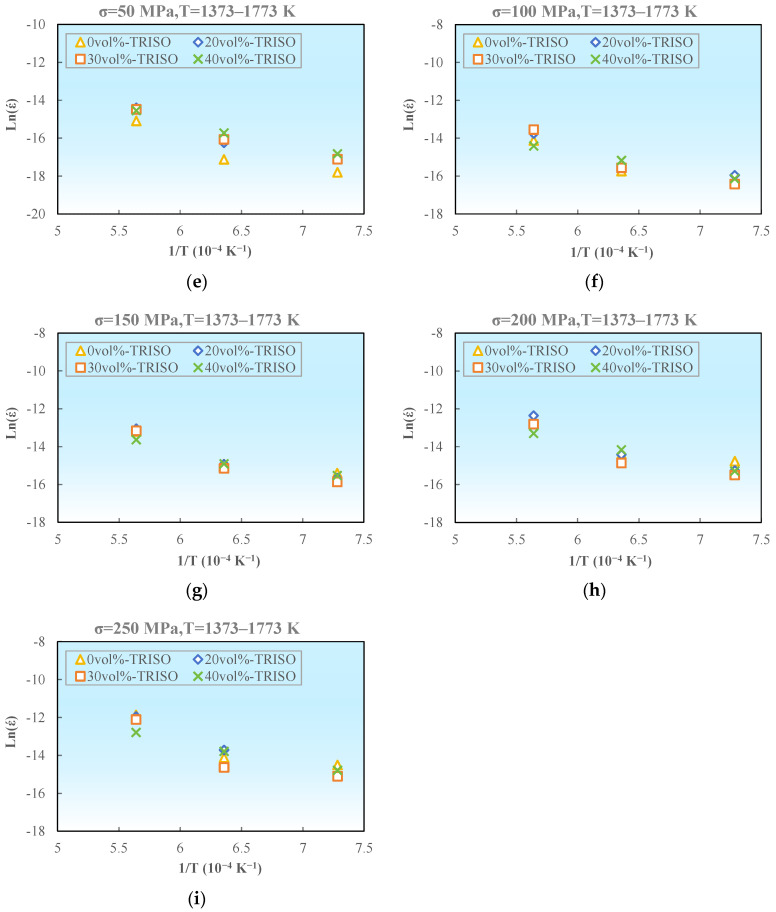
Relationship between creep rate and creep temperature, ln (*έ*)-1/T: (**a**) σ = 5 MPa, T = 1873–2073 K; (**b**) σ = 20 MPa, T = 1873–2073 K; (**c**) σ = 40 MPa, T = 1873–2073 K; (**d**) σ = 50 MPa, T = 1873–2073 K; (**e**) σ = 50 MPa, T = 1373–1773 K; (**f**) σ = 100 MPa, T = 1373–1773 K; (**g**) σ = 150 MPa, T = 1373–1773 K; (**h**) σ = 200 MPa, T = 1373–1773 K; (**i**) σ = 250 MPa, T = 1373–1773 K.

Based on the aforementioned experimental data, the steady-state creep strain rate of pellet samples with different TRISO contents were logarithmically calculated and plotted as ln ($\dot{\varepsilon }$)–1/T, as demonstrated in Figure 10. The slope of the curve reflects the creep activation energy *Q*. As shown in Table 4 and Table 5, the creep activation energies under high temperature low stress and low temperature high stress are calculated respectively.

**Figure 11:**
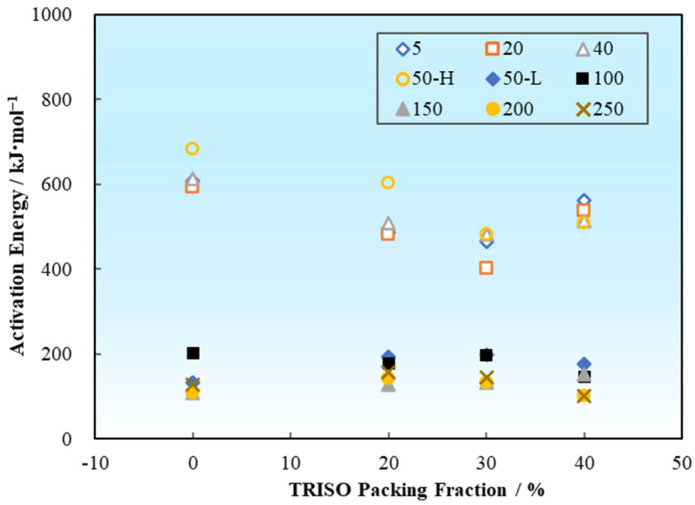
Variation of creep activation energy with TRISO particle packing fraction.

Temperature and stress are crucial factors affecting the creep activation energy of materials. Changes in temperature will affect the creep activation energy and diffusion activation energy, thereby altering the creep mechanism and properties. In the case of high stress creep, there will be a higher creep rate and lower activation energy, however, a lower creep rate and higher activation energy would emerge when the stress decreases. The tendency of average creep activation energies of the pellet samples varies with the TRISO content as shown in Figure 11. For the high temperature low stress cases (1873–2073 K, 5–50 MPa), the average creep activation energy of the 0 vol% TRISO pure matrix pellet is *Q*_0 vol% TRISO, high temperature low stress_ = 623.77 kJ/mol. The introduction of TRISO particles slightly reduces the creep activation energies of the dispersed fuel pellet (*Q*_20–40 vol% TRISO, high temperature low stress_ = 457.81–529.98 kJ/mol). Considering the statistical error of the experimental data, the creep activation energy is at the same level when the TRISO particle content is between 20 and 40 vol%. For the low temperature high stress cases (1373–1773 K, 50–250 MPa), the creep activation energies are significantly lower compared to the high temperature low stress cases (*Q*_0–40 vol% TRISO, low temperature high stress_ = 135.14–161.59 kJ/mol).

For ZrC-30 wt% SiC composite, the stress exponent at 1550 °C for stresses ranging from 60 to 100 MPa is 3.1 ± 0.2, the activation energy at a stress of 60 MPa for temperatures ranging from 1500 to 1550 °C is 848 ± 132 kJ/mol [29]. Correspondingly, the ZrC-46 wt% SiC matrix in this article has a creep stress exponent of 1.14 and a creep activation energy of 623.77 ± 40.97 at 1600 °C. The activation energy of the composite is closer to that of ZrC, which is 632 ± 52 kJ/mol [30]. Moreover, investigations on creep properties of high entropy carbide ceramics (HEC) were performed under the conditions of 1400–1600 °C, with a resulting stress exponent of n = 2–3 and creep activation energy *Q* = 170–212 kJ/mol [32,33].

### 3.4. Creep Modelling

The behaviors of dispersed coated particle fuel during in-pile operation are affected by complicated multi-physical factors such as temperature, stress, irradiation, etc. In order to evaluate mechanical behavior, a corresponding creep model needs to be established. This study focuses on the impacts of temperature, stress, and TRISO packing fraction on the pellet creep performance. Based on the experimental results, a creep model for dispersed fuels is established, providing a reference for subsequent research on fuel behavior mechanisms and performance evaluation.

**Figure 12:**
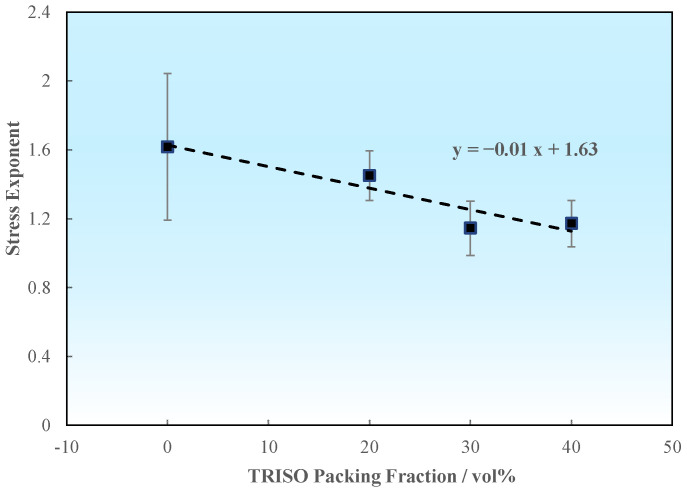
Fitting relationship between average stress exponent and TRISO packing fraction.

Based on the previous fitting, the relationship between stress exponent and TRISO packing fraction could be determined by Figure 12, as follows:

(4) n=−0.01·PF+1.63

where *PF* is the packing fraction of TRISO in pellet, %.

**Figure 13:**
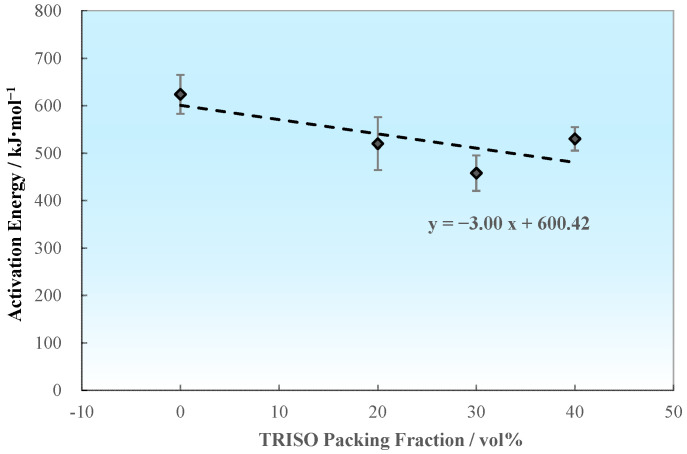
Relationship between average creep activation energy Q and TRISO packing fraction (high temperature and low stress).

Similarly, the relationship between creep activation energy and TRISO packing fraction can also be further examined by taking the average value. According to Section 3.3.2, the creep activation energies under low temperature and high stress conditions are almost unaffected by the packing fraction of TRISO, with an average value of approximately 148.36 kJ/mol. Under high temperature and low stress conditions, the average creep activation energies slightly decrease with the decrease in TRISO packing fraction, as shown in Figure 13. The expression of how creep activation energy varies with TRISO packing fraction is as follows:

(5) $$\begin{array}{c} Q=148.36,\;T\leq 1773\;K\\ Q=-3.0\cdot PF+600.42,\;T>1773\;K \end{array}$$

According to Equation (1),

(6) $$A=\dot{\varepsilon }{\cdot \sigma }^{-n}\cdot exp\left(\frac{Q}{kT}\right)$$

By substituting the values of *n* and *Q* from Equations (4) and (5) into Equation (6), combined with the creep experimental results, the model coefficient *A* can be obtained as follows:

(7) $$\begin{array}{c} A=-3.09\times 10^{-9}\cdot PF+3.38\times 10^{-7},\;T>1773\;K\\ A=2.93\times 10^{-11}\cdot PF+2.37\times 10^{-10},\;T\leq 1773\;K \end{array}$$

Therefore, by combining Equations (1), (4)–(7), the steady-state creep rate calculation model for dispersed coated particle fuel pellets can be obtained as follows:

(8) $$\begin{array}{c} {\dot{\varepsilon }}_{HT}=\left(3.38\times 10^{-7}-3.09\times 10^{-9}\cdot PF\right)\\ \sigma^{\left(1.63-0.01\cdot PF\right)}exp\left(-\frac{600.42-3\cdot PF}{kT}\right),\;T>1773\;K\\ {\dot{\varepsilon }}_{LT}=\left(2.37\times 10^{-10}+2.93\times 10^{-11}\cdot PF\right)\cdot \sigma^{\left(1.63-0.01\cdot PF\right)}exp\left(-\frac{148.36}{kT}\right),\;T\;\leq 1773\;K \end{array}$$

where the steady-state creep strain rates at high and low temperatures are denoted as ${\dot{\varepsilon }}_{HT}$ and ${\dot{\varepsilon }}_{LT}$, respectively, s^−1^; *σ* is the creep stress, MPa; *PF* is the packing fraction of TRISO particles, %; *k* is the Boltzmann constant, 8.314 kJ/mol/K; *T* is the creep temperature, K.

As the TRISO packing fraction increases, the creep activation energy *Q* slightly decreases, which leads to an increase in the creep strain rate. However, the creep model constant *A* is also related to the packing fraction and decreases with increasing packing fraction, resulting in a decrease in the creep strain rate. The combined effect of *Q* and *A* is manifested as an overall decrease in the creep strain rate as the TRISO packing fraction increases.

## 4. Conclusions

Compressive creep experiments were conducted using a ZrC–SiC composite matrix with dispersed coated particle surrogate pellets comprising different TRISO particle packing fractions under temperature ranges of 1373–2073 K and stress ranges of 5–250 MPa. The creep behaviors and mechanisms of the dispersed coated fuel pellets were elucidated, and key parameters such as creep stress exponents and creep activation energies were obtained as a function of TRISO packing fraction. Additionally, a creep model was established, which provides a valuable reference tool for subsequent research and application of dispersed coated particle fuels.

(1) The creep deformation of coated particle dispersed fuel pellets was mainly activated by the ZrC–SiC matrix, while the TRISO particle structure remained basically unchanged. The introduction of TRISO particles enhanced the creep performance of the pellets.
(2) Creep stress exponents of the dispersed pellet ranged from 0.89 to 2.12, creep activation energies ranged from 457.81 to 623.77 kJ/mol for the high temperature low stress cases (1873–2073 K, 5–50 MPa), and from 135.14 to 161.59 kJ/mol for the low temperature high stress cases (1373–1773 K, 50–250 MPa). The creep stress exponents and creep activation energy of the dispersed pellet changed slightly with TRISO packing fraction.
(3) Based on the experimental results, a steady-state creep strain rate calculation model for dispersed fuels is established, providing a valuable reference tool for the research and deployment of ceramic matrix dispersed coated particle fuels.

## Figures and Tables

**Table 1:** Densities of pellet samples.

TRISO Packing Fraction, vol%	Density, g/cm^3^	Theoretical Density, g/cm^3^	Relative Density, %	TRISO Packing Fraction, vol%
0	4.30	4.32	99.5	0
20	3.92	3.96	99.0	20
30	3.62	3.78	95.8	30
40	3.51	3.60	97.5	40

**Table 2:** Compressive creep test matrix.

Samples	Stress/MPa	Temperature/K					
		1373	1573	1773	1873	1973	2073
		Holding Time ~60 min			Holding Time ~10 min		
TRISO packing fraction 0 vol%, 20 vol%, 30 vol%, and 40 vol%	5				√	√	√
	20				√	√	√
	40				√	√	√
	50	√	√	√	√	√	√
	100	√	√	√			
	150	√	√	√			
	200	√	√	√			
	250	√	√	√			

**Table 3:** Creep stress exponents of pellet with different temperatures and TRISO contents.

	Temperature	1373 K	1573 K	1773 K	1873 K	1973 K	2073 K
TRISO Content							
0 vol%		2.12	1.89	1.98	1.14	1.26	1.27
20 vol%		1.20	1.51	1.54	1.49	1.37	1.60
30 vol%		1.24	0.89	1.36	1.07	1.16	1.13
40 vol%		1.23	1.17	1.09	1.36	0.95	1.27

**Table 4:** Creep activation energies of pellets with different TRISO content (high temperature low stress) (Unit: kJ/mol).

	Stress	5 MPa	20 MPa	40 MPa	50 MPa	Average (Std. Deviation)
TRISO Content						
0 vol%		606.85	592.17	612.14	683.91	623.77 (40.97)
20 vol%		487.40	482.50	507.84	602.15	519.97 (55.88)
30 vol%		465.15	402.83	481.59	481.67	457.81 (37.47)
40 vol%		561.88	536.60	513.85	507.60	529.98 (24.65)

**Table 5:** Creep activation energies of pellets with different TRISO content (low temperature–high stress) (Unit: kJ/mol).

	Stress	50 MPa	100 MPa	150 MPa	200 MPa	250 MPa	Average (Std. Deviation)
TRISO Content							
0 vol%		133.50	202.81	108.50	107.70	129.15	136.33 (38.96)
20 vol%		193.87	178.17	128.91	143.23	157.64	160.36 (26.13)
30 vol%		197.80	198.20	133.14	132.85	145.94	161.59 (33.66)
40 vol%		175.82	147.07	151.82	100.43	100.58	135.14 (33.45)

## Data Availability

The original contributions presented in this study are included in the article. Further inquiries can be directed to the corresponding author.
